# The influence of memory on indoor environment exploration: A numerical study

**DOI:** 10.3758/s13428-015-0604-1

**Published:** 2015-07-14

**Authors:** Vaisagh Viswanathan, Michael Lees, Peter M. A. Sloot

**Affiliations:** 1 TUM CREATE Limited, 1 CREATE Way, #10-02 CREATE Tower, Singapore, 138602 Singapore; 2 School of Computer Engineering, Nanyang Technological University, 50 Nanyang Avenue, Singapore, 639798 Singapore; 3 Computational Science, University of Amsterdam, Amsterdam, The Netherlands; 4 Complexity Institute, Nanyang Technological University, Singapore, Singapore; 5 ITMO University, St. Petersburg, Russia

**Keywords:** Indoor navigation, Exploration, Serious games, Markov models, Memory, Agent based modeling

## Abstract

Understanding human behavior in the context of exploration and navigation is an important but challenging problem. Such understanding can help in the design of safe structures and spaces that implicitly aid humans during evacuation or other emergency situations. In particular, the role that memory plays in this process is something that is crucial to understand. In this paper, we develop a novel serious game-based experimental approach to understanding the non-randomness and the impact of memory on the human exploration process. We show that a simple memory model, with a depth of between 6 and 8 steps, is sufficient to approximate a ‘human-like’ level of exploration efficiency. We also demonstrate the advantages that a game-based experimental methodology brings to these kinds of experiments in the amount of data that can be collected as compared to traditional experiments. We feel that these findings have important implications for ‘safety-by-design’ in complex infrastructural structures.

## Introduction

Traditionally, experiments on human way-finding involve participants performing way-finding in a real environment. In some cases, this would be followed by tasks that test the knowledge that they developed (Siegel & White, 1975; Ishikawa & Montello, 2006; Moeser, 1988). In experiments aimed at studying how exploration is done, the video of player movement would often be analyzed (Hölscher et al. 2006). The nature of the data necessary, namely spatio-temporal tracks of many individuals, lead to virtual environments having certain advantages over real environments due to the ease of recording movement and the reduced overhead of conducting experiments (Difonzo et al. 1998; Davis et al. 2009; Loomis et al. 1999; Stankiewicz et al. 2006). There have also been studies (ONeill, 1992; Montello et al., 2004) comparing performance in virtual worlds and real-world experiments, showing that human behavior in simulated environments is a good approximation of real-life behavior.

Recent years have seen the emergence of serious games as an approach to conducting human subject experiments (Pengfei et al. 2011; Michael & Chen, 2005; Waller et al. 2002). The enjoyability and ubiquity inherent in games (Pengfei et al. 2011), tend to attract more participants than traditional virtual environment-based experiments. Bode and Codling (2013) developed a point-and-click game to study exit route choice during evacuation and obtained more than 150 participants for their experiment. In the past this type of approach has been used for validating crowd simulation models (Pelechano et al. 2008; Pengfei et al. 2011; Viswanathan et al. 2014) and understanding behavioral responses to dynamic information during egress (Bode et al. 2014; Viswanathan & Lees, 2012). Serious games (Michael & Chen, 2005) have a long history in training, for example, aircraft simulation (Hays et al. 1992) and medical training (Barry Issenberg et al. 1999). In this paper, we plan to use a game for exploring and understanding the role of memory in way-finding in indoor environments.

Tolman (1948) is credited with coining the term *cognitive map* to refer to the internal representation of the environment used by rats for exploration. Lynch (1960), subsequently, proposed that the human cognitive map is composed of five components: paths, edges, districts, nodes, and landmarks. Since then, several studies have explored the central role played by landmarks and routes between these landmarks in the initial cognitive map and the time taken by people to develop somewhat complete knowledge of the layout, also known as *survey knowledge* (Siegel and White, 1975; Ishikawa & Montello, 2006; Moeser, 1988). Thorndyke and Hayes-Roth (1982) discovered that survey knowledge can be developed very quickly (≈20 minutes) when maps are used. However, without the use of maps and compasses, if knowledge is gained through actual daily movement through the environment then survey knowledge may take up to 2 years to develop. This was further demonstrated by Moeser (1988) who found that people generally formed an image of the layout of the building within the first month while later experience just extends this in minor ways. Thus, the exploration strategies used by new occupants have a significant effect on the cognitive maps eventually formed.

Way-finding in indoor environments is influenced by several factors. Gibson’s work on information design for public spaces discusses the different strategies to help way-finding based on the purpose and cultural context of buildings (Gibson, 2009). Research has also revealed the different factors like number of choice points, visual access, degree of architectural differentiation, etc., that determine way-finding difficulty in an environment (Gopal et al., 1989; Best, 1970; Weisman, 1981; Gärling et al., 1983; Evans & Pezdek, 1980). Hölscher et al. (2006) recorded videos of people exploring multi-floor buildings in order to investigate way-finding strategies. They discovered three major strategies of which the *floor-first strategy* was observed to be the most efficient and the one used by more experienced participants. As the name suggests, this strategy involved the person trying to get to the required floor first and then exploring horizontally to find the goal.

Kuipers (1978) developed the highly influential TOUR model of spatial knowledge processing for *large-scale urban spaces*. He proposed *the route skeleton*-based model of exploration (Kuipers et al. 2003), which was also observed by Hölscher et al. (2006) in a few participants. He chose to use *desktop virtual reality* experiments for validation which could, in theory, analyze the spatio-temporal patterns of participants in more detail than experiments of the kind conducted in Hölscher et al. (2006). Virtual environments have also been used to demonstrate the importance of structural landmarks and to investigate exploration in unknown environments through comparison against an *ideal-navigator model* (Stankiewicz and Kalia, 2007). They explored the inefficiencies in human navigation in unknown environments by comparing against an ideal navigator, i.e., one that has *perfect* perceptual processing, *perfect* map memory, and the *ideal* decision strategy. The agent-based analysis discussed in the present paper uses a similar approach.

Existing virtual environment-based experiments (Stankiewicz et al. 2006; Kuipers, 1978) use long-lasting experiments that are rather difficult to administer to more than ten participants. Generally, the enjoyability (for the participant) of the virtual environment is not taken into consideration when developing these experiments. Game-based experiments have been shown to add value in terms of participant engagement (Berger et al. 2000; Connolly et al. 2012; Difonzo et al. 1998; Washburn, 2003; Hawkins et al. 2013). In designing game-based experiments, it is also important to realize and acknowledge some of the limitations of the methodology as well (Washburn, 2003; Hawkins et al. 2013; Donchin, 1995). For example, Hawkins et al. (2013) argue that added enjoyability does not add or remove significantly from the quality of the results produced. However, we believe that the added enjoyability of the game will help obtain a larger (and more diverse) dataset that allows for novel analyzes of the sort that has traditionally not been possible even in virtual environment-based experiments.

Virtual environment-based experiments provide a degree of control of experiment design that is much more difficult to realize in real-world experiments (Difonzo et al. 1998). However, it can be argued that this comes at the cost of considering spatial cognition as a disembodied process (Wilson, 2002; Difonzo et al. 1998). However, this is not entirely true because, in a game (especially of the kind proposed in this paper), the players can interact with the environment around them, thus taking into consideration the embodied nature of cognition.

Montello et al. (2004) suggest that the immersiveness of the virtual environment is an important factor in its effectiveness. Virtual reality (VR) headsets provide a way to address the issue of embodied cognition by allowing the creation of virtual environments that allow for experimental control without considering cognition as a disembodied process. However, creating these kinds of immersive VR experiments (Waller et al. 2007; Stankiewicz and Eastman, 2008) require significantly more investment in terms of development effort and financial cost (Dahmani et al. 2012; Durgin & Li, 2010; Bowman & McMahan, 2007; Washburn, 2003). Studies comparing head-mounted VR experiments against desktop-based VR have failed to show significant differences in results produced (Dahmani et al. 2012).

In this paper, we adopt a *desktop virtual reality-based game* to gain an understanding of how humans, with no knowledge of an environment, explore. We do this by developing a novel way of identifying the role that memory and non-randomness plays in human exploration. The method involves experiments in which participants play an exploration game; in the game, they are asked to explore a multi-floor building and complete a set of tasks within a certain time limit. All the movement and actions of the players are logged and later analyzed for patterns. We also develop a novel way of identifying the role that memory and *non-randomness* play in human exploration from the data extracted. Thus, the main motivations of this paper, and therefore its main contributions, are:

1. The development of a novel and scalable game-based methodology to study indoor way-finding behavior.
2. The demonstration of such a scalable method’s usefulness in determining how memory influences exploration efficiency and an individual’s ability to navigate within an environment.
3. Determine the influence of memory on exploration efficiency and an individuals ability to navigate within an environment.

## Setup of the experiment

Way-finding experiments in virtual reality environments consist generally of two parts: knowledge acquisition and task performance based on this knowledge. Most existing experiments try to control the knowledge acquisition part of way-finding. A typical example is (Meilinger et al. 2008) where the author ensured that all participants received identical stimuli in order to be able to fairly compare their task performance based on this knowledge acquisition. Participants were asked to watch a video rather than actively navigate through the environment. This has been the typical approach to date. We believe, however, that the paths taken during exploration could reveal interesting aspects of human exploration (Dahmani et al. 2012).

In order to test this hypothesis and understand more about the way in which humans explore environments and store spatial knowledge, we created a game that requires both exploration and way-finding and analyzed how the game was played. In creating the environment, we strived to ensure that it had enough complexity and diversity to be engaging and invite exploration (Kaplan & Kaplan, 1982; Montello et al. 2004).

### The game

**Fig. 1:**
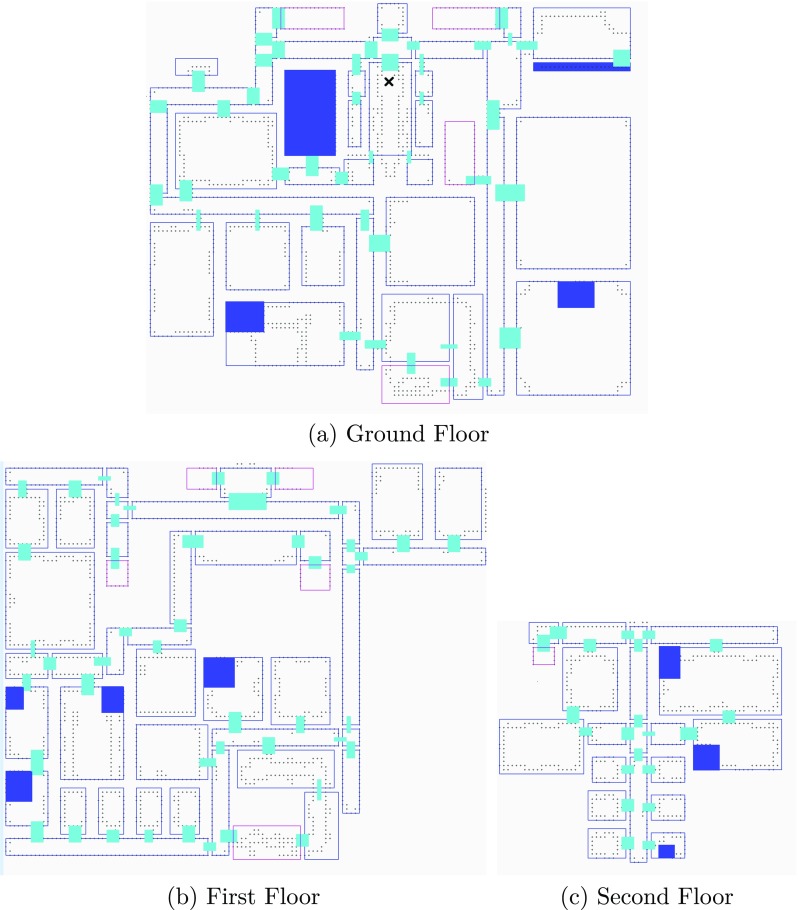
Floor plans of the three floors. The *X* indicates the starting point. The *blue color* indicates the checkpoints that have to be reached by the participant during exploration (this is a modified version of an existing Minecraft map [ http://www.planetminecraft.com/project/royal-palace/])

Figure 1 shows the layout of the environment that the game is played in. The player starts on the ground floor at the point indicated by X. During the first phase of the game, called the *exploration phase*, the player performs an unguided exploration of the three-floor 44-room environment. The exploration phase finishes when the player has found all the checkpoints that are distributed over the three-floor environment. The checkpoint locations are chosen in a way that ensures that roughly 90 % of the environment would have been visited by the player by the end of the exploration phase (confirmed by game statistics in Table 2). The *knowledge testing* phase follows the exploration phase. During this phase, the player has to sequentially execute three tasks in three locations, one on each floor. The players do not have access to maps or directional signs to help them navigate. However, simple text-based *identification signs* are placed in front of each room to identify the name, and at staircase entrances and exits to identify the floor number. Without these, the environment would be too confusing for players to navigate (Gibson, 2009). A more detailed description of the game and the environment is given in Appendix B.

### Participants

Fifty young adults (14 women, 36 men, age range 18–30 years) were recruited with fliers posted on the Nanyang Technological University campus. Participants were compensated $5 for their participation in the game. It was played by all participants on the same computer in a lab with minimal distractions.

Participants were given 5 min to familiarize themselves with the controls of the first-person game, which involved using the keyboard direction keys for movement and the mouse for altering the move and view direction. A mouse click would allow the participant to interact with the environment by either opening doors or freeing prisons. The participants were given 45 min to complete the game. Of the 50 participants, the data from only 44 participants were used, the remaining six (one woman, five men) experienced motion sickness from the movement in the first-person gaming environment and had to quit playing before the game could be completed. Next, we explain the way in which the data was analyzed.

## Analysis

**Fig. 2:**
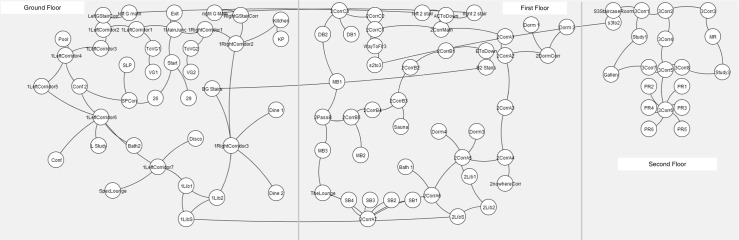
This figure shows a graphical representation of the room layout in Fig. 1

Figure 2 shows the room layout show in Fig. 1 as an undirected graph. A directed *movement graph* is calculated for each player, with the edges of the graph indicating the direction and time of movement from one node to the next. The analysis presented in the following sections is performed using these graphs.

### Types of exploring agents

The motivation of our analysis is to determine how memory influences exploration efficiency and an individual’s ability to navigate within an environment. In order to do this, we use an approach similar to the ideal navigator model of Stankiewicz et al. (2006). We compare the movement of the players to different types of simple exploring agents inhabiting the same environment as the player. Through this comparison, we determine the ways in which a player is different from, or similar to, each of these agents.

We compare the *movement graph* of the players to three kinds of exploring agents: unbiased random walkers, agents with perfect *m*-step memory, and Markov agents. Just like the actual players, these agents have no prior knowledge and explore the undirected graph shown in Fig. 2. Our analysis is done based on four sets of directed movement graphs:

- *Actual players*: This is the set of 44 player movement graphs, i.e., the movement graphs introduced at the beginning of this section.
- *Unbiased random walker*: The next node to which this agent moves is chosen randomly with equal probability. Each iteration of the random walk is performed until the walker covers 100 % of the environment. A collection of random walks is obtained until the variance in the radius of gyration of generated movement graphs stabilizes. The radius of gyration is the standard deviation of a walker’s position to the center of mass (centroid) of movement (Cheng et al. 2011). It gives a measure of the locality of the graph.
- *Agent with perfect m-step memory*: This is a biased random walker with an *m*-step memory. It moves exactly like the random walker except that it avoids moving back to any of the *m* rooms it visited previously. If there is no unvisited room, the agent checks its *m*-step memory for an unexplored junction. If such a junction exists, it goes back to that point and continues exploring in an unvisited direction. If such a junction does not exist, the agent simply chooses, at random, a neighboring location to the current node with equal probability. Depending on the calculation being performed, the path is generated for a specific number of hops or until a specific coverage is achieved. The coverage is simply calculated as:
  1 $$ \text{coverage} = \frac{\text{number of rooms visited}}{\text{total number of rooms}} \times 100  $$
  A total of 30,000 such movement graphs are generated for each value of *m* for each experiment. This was empirically found to be a good enough value to ensure minimal variation in the radius of gyration.
- *m* ^*th*^ *-order Markov agents*: These agents mimic the movement of the average of the ensemble of players assuming they had only an *m*-step memory. The action of an *m* ^*th*^-order Markov agent at a particular point in the path is a function of the actions of the actual players who had taken the same *m* steps. This is explained in more detail in the next section. The calculations are performed for *m* from 1 to 13. As with the agent with perfect memory, the movement graphs are generated either for a specific number of hops or until a specific amount of coverage is achieved. Also, as before, 30,000 movement graphs are generated for each value of *m*.

Simulations of movement were used to generate movement graphs for each of these types of agents and the results of this analysis are presented in the Results section. Before this, the next section explains the working of the Markov agents in more detail.

### Markov agents

In this section, we present how the movements of the Markov agents are calculated. The chief motivation of this Markovian analysis was to investigate the role that memory plays in the exploration of the environment (Refer to Table 1 for a summary of symbols used in this section and their meaning).

**Table 1:** Summary of symbols and their meaning

Symbol	Meaning
*r*	A particular node
*P* ^(*n*,*D*)^	List of all paths of length *n* taken by individuals
	in dataset *D*
*q* ^(*n*)^	A particular path of length *n*
$P^{(n,D)}_{q^{m}}$	List of all paths in *P* ^(*n*,*D*)^ where the first *m* steps
	are represented by *q*

We take an *m* ^*th*^-order Markov model to represent an *m*-step memory of the explorer, where steps constitute node visits on the undirected graph (Fig. 2). One way to speculate on the size of the memory used by a human during exploration is to predict a path of length *n* from some Markov data of order *m*<*n*. We hypothesize that *if the movement of the actual players can be predicted using an* *m* ^*th*^ *-order Markov agent, then this implies that humans use a working memory of size m steps during exploration.*

In a general *m* ^*th*^-order Markov process, the basic idea is that the action at any point of time depends only on the previous *m* actions. By stating that the process of exploration is an *m* ^*th*^ order Markov process, we assume that the next node that is visited by a player is only dependent on the previous *m* steps. This is different from an agent with *m*-step memory that tries to avoid the previous *m* nodes. Since the next step is dependent on the actions of players who have visited that same subsequence of *m* nodes, the Markov model theoretically encapsulates other factors like layout, visibility, and so forth, and thus, unlike the former, has *imperfect recollection*.

There are several methods for estimating the order of different kinds of Markov models (Strelioff & Crutchfield, 2014; Papapetrou & Kugiumtzis, 2013; Akaike, 1998; Schwarz, 1978) and especially for Markov-chain models of the sort used in this paper (Papapetrou & Kugiumtzis, 2013; Akaike, 1998; Schwarz, 1978). However, these methods tend to perform much worse when the state space of the Markov model is large (Papapetrou & Kugiumtzis, 2013) like in the present scenario (44 rooms). Singer et al. performed a similar analysis on online navigation behavior to determine people’s browsing habits (Singer et al. 2014). The key to being able to do this analysis was abstracting away from specific page transitions (a very large state space) and studying memory effects on a topical level by representing click streams as sequences of topics or categories (state space of less than 10). In the absence of a method of effectively categorizing rooms based on some reliable criteria, the existing methods become unreliable for order estimation. Thus, we use the data available to simulate exploration through an agent-based model and compare results against the different models as explained in the previous section.

**Fig. 3:**
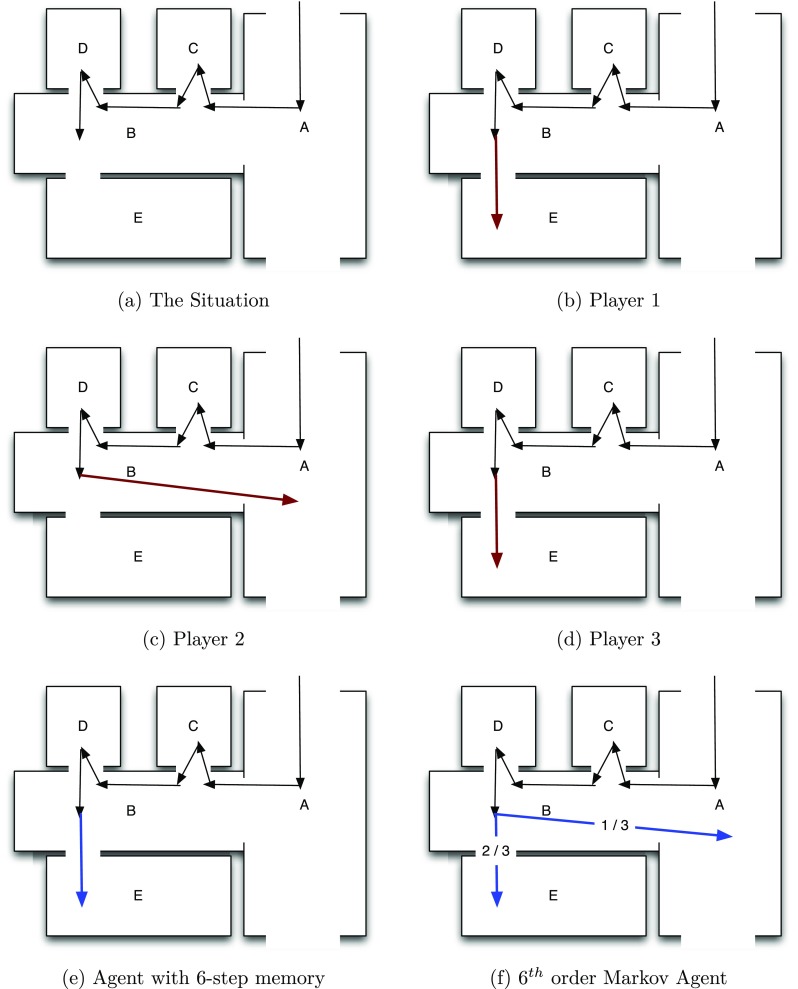
Given the situation in **a**, the agent with six-step memory would move next to room E since it has the list B, D, B, C, B, A as the six rooms visited immediately previously. However, the action of the 6^*th*^-order Markov agent in this situation is dependent on the actions of the players (**b**, **c** & **d**) who happened to be in the same situation. Since one out of the three players who were in the given situation next moved to room A, there is a 1/3 chance that the Markov agent will visit room A next

Figure 3 explains the idea of a Markov agent by contrasting its behavior with that of an agent with simple *m*-step memory. In the example layout—with the rooms C, D, and E connected to corridors A and B—we consider the situation where the exploring agent has moved from A to B to C to B to D and back to B. Assuming a six-step memory, the simple memory agent would definitely visit room E since all other rooms have been visited in the last six steps. However, the 6^*th*^−*o* *r* *d* *e* *r* Markov agent’s action in this situation depends on the actions of the actual players in this situation. Since two out of the three players visited room E and one out of the three players visited room A, the next step of the Markov agent can either be room E or A with 2/3 and 1/3 probability, respectively.

More generally, for an *m* ^*th*^-order Markov agent, given a particular path of length *m*, the (*m*+1)^*th*^ step is generated using the previous *m* steps, that is, the 1^*st*^ to *m* ^*th*^ step. Let the set of all paths of length *m*+1 in the dataset *D* be represented by *P* ^(*m*+1,*D*)^ and let *q* be the traversed path of *m* steps. First, we select the subset of *P* ^(*m*+1,*D*)^ whose first *m* steps are the same as *q*. If this is represented as $P^{(m+1,D)}_{q^{m}}$, then the probability of the (*m*+1)^*th*^ step being a particular room *r* is the proportion of paths in $P^{(m+1,D)}_{q^{m}}$ with the (*m*+1)^*th*^ step arriving at *r*.

Following this, the (*m*+2)^*th*^ step can be predicted by doing the same calculation using the preceding *m* steps from 2 to *m*+1. This process can be continued to generate a directed movement graph of *n* edges using just the *m* ^*th*^-order Markov data. As mentioned previously, calculations are done using 30,000 paths generated like this. In the cases where $P^{(m+1,D)}_{q^{m}} = \emptyset $, the (*m*−1)^*th*^-order probability is taken to determine the destination.

The validity of this approach with respect to the size of the dataset available is presented in Appendix A. Next, we look at the results of our analysis.

## Results

From the game, the complete movement traces of 50 players were obtained. Some general statistics on the data are presented in Table 2. The movement data obtained was analyzed by comparing against agents of different types to determine patterns and gain a better understanding of the movements of the players. This section presents the results of three kinds of analysis that were performed. First, we present the results of a simple check of the frequency of visits to each room. Following this, we compare the efficiency of movement of the players against the different agents. Finally, we present some empirical observations from the movement graphs of the actual players.

**Table 2:** List of some general game statistics. The coverage and hop count give an estimate of exploration efficiency

Phases	Exploration	Knowledge testing
Duration	25.15±5.70 minutes	3.85±1.03 minutes
Coverage	89±1 %	45±8 %
Hop Count	252.6±7.5	88±26

Exploration coverage of roughly 90 % meets the design objectives discussed in the section describing the game

### Room visit frequencies

In our first experiment, we calculate the frequency of visits for each room per player and compare this against the unbiased random walker. This is a simple test to determine if the players have a pattern or strategy in their exploration that is different from a random walk. If there is a significant difference in the number of times a particular floor or area of the building is visited by a player then this will be revealed by this comparison against an unbiased random walker.

For each room *r* we calculate the normalized number of visits *α*. For any room *r*, let *f*_*p*_(*r*) be the total number of times room *r* is visited by the player *p* and let *f*_*rw*_(*r*) be the number of times the average random walker visited the same room. We define the normalized number of visits *α* as:

2 $$ \alpha_{x}(r) = \frac{f_{x}(r)}{\sum\nolimits_{a \in R} f_{x}(a)}  $$

This is calculated for both the player and the random walker. Using this the visit ratio, *y*_*p*_(*r*), of the actual player is calculated for each room as:

3 $$ y_{p}(r) = \frac{\alpha_{p}(r)}{\alpha_{rw}(r)}  $$

**Fig. 4:**
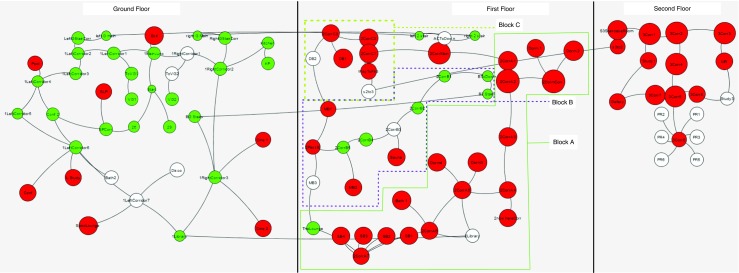
Figure 2 scaled by normalized number of visits as described in Eq. 3. *Red* color indicates a *y* value of greater than 1.05 and the *green* color indicates a value of less than 0.95. The diameter of each node in this graph is scaled to *y*_*r*_×(*u* *n* *s* *c* *a* *l* *e* *d* *d* *i* *a* *m* *e* *t* *e* *r*)

A random walker’s frequency of visits to a particular node is purely a function of the topography of an environment, i.e., the nodes and their connectivity. Thus, unlike *α*_*p*_(*r*), *y*_*p*_(*r*) would not contain the effects of the degree of a node. This means that if a room *r*_1_ has lower value of lower *y*_*p*_(*r*) than *r*_2_, this is not because of the room having lesser connectivity. The average *y*(*r*) over the ensemble of player data is then calculated. This is illustrated in Fig. 4. This figure shows the value of *y*(*r*) of each room as a scaled version of Fig. 2. The red color indicates those rooms that have 5 % more visits in the human data than the random walker data and the green color indicates those that have 5 % less visits than a random walker; the node is colored white otherwise. The diameter of each node in this graph is scaled by *y*_*r*_.

Therefore, a white color indicates that the normalized number of visits in both the *random walker* dataset and in the *actual player* dataset are within 5% of each other. A value greater than 1 indicates that players visited the room more than the random walker and a smaller value indicates the opposite. There is a marked difference in the visits by the players and visits by the random walker. It can also be seen that the average number of visits on the second floor was higher than the number of visits on the first floor, which was more than the number of visits on the ground floor.

**Fig. 5:**
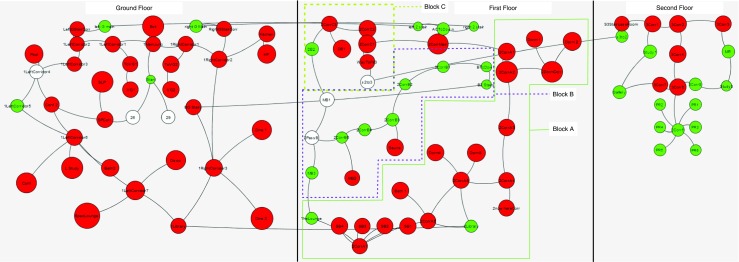
Figure 4 normalized to floor instead of the total number of visits. The fact that this graph is different from Fig. 4 indicates that, unlike a random walker, a player differentiates between a simple link between rooms or corridors and a staircase that links two floors

Despite a random walker not differentiating between staircases and other links, a random walker might have more visits to one floor than another purely because there are fewer inter floor connections than other links. For example, floor 3 has only one staircase that leads the random walker out of the third floor. To confirm that the inter floor differences for the actual player is not because of this, we normalize Eq. 2 to the number of visits on the floor rather than the total number of visits. On doing this, if the graph is different form Fig. 4, it would imply that unlike a random walker, a player differentiates between a simple link between rooms or corridors and a staircase, which is a link between floors. Figure 5, which is the floor normalized version of Fig. 4 does clearly show this.

Figures 4 and 5 provide a possible validation of a variation of the floor-first strategy (Hölscher et al. 2006) used for exploration. The strategy in the original paper was for way-finding with a particular goal, but here it seems to be being used for exploration (which is way-finding without a definite goal). The players seem to consider each floor as a separate entity and are generally reluctant to take the staircase. This might also be because the process of separating each floor helps in bringing some organization and structure to the confusing room layout and the process of exploration (that is, completely explore one level before the next).

There is a chance that this aversion to taking staircases is a consequence of the structure of the game environment or the controls in the game. This would happen if the staircases were difficult to find or climbing staircases required different controls from moving through doors or walking through corridors. However, this is not the case in the designed game. The player just has to press the forward button to move in the environment regardless of whether it is a staircase or a corridor or a room. Also, the staircases are clearly visible from the neighboring nodes with sign boards further confirming their location.

The hypothesis of the existence of this floor-first strategy is further strengthened by the low visit frequencies to Block B on the second floor Fig. 5. There are four staircases that take a player from floor 1 to floor 2: two of these lead to Block A, one to Block C, and one to Block B. Block A and C are directly connected by a corridor whereas the only paths to Block B from the same floor are through rooms DB2 and The Lounge in Block A and C, respectively. This means that unlike Block A and C, Block B is not accessible via any direct corridor from the same floor. Since people show a clear inclination to exploring through corridors as shown in the Empirical Analysis Section of this paper, the only obvious way to access Block B is by going down a floor. The fact that Block B has fewer visits seems to suggest that people resist going down a floor, thus further strengthening the hypothesis of a floor-first strategy in exploration.

### Expected coverage given number of hops

**Fig. 6:**
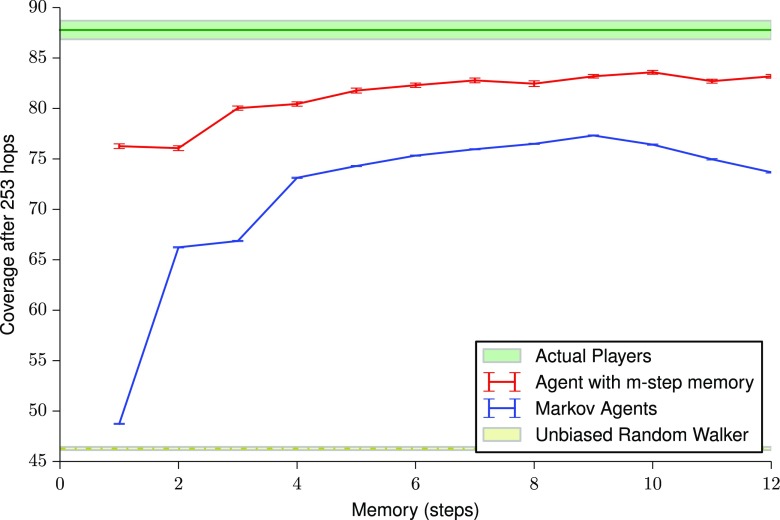
Standard error plot of the average coverage after 253 hops as a function of memory size. The low values of standard error on the agent paths are because these calculations are calculated over several thousand paths that are required for the radius of gyration to stabilize. A value of 253 hops was taken because this was the average number of hops taken by a player

The average coverage after a given number of hops gives an estimate of the efficiency and effectiveness of exploration. On average, each player took 252.6±7.5 hops during their exploration phase. The coverage achieved by the other types of agents after 253 hops was calculated by generating the required set of movement graphs as explained earlier. Figure 6 shows the results of this calculation.

The figure seems to indicate that even a second-order Markov agent, that is one whose next position is only dependent on its current and previous position, performs much better than an unbiased random walker performs. It also seems to indicate that after 253 hops the performance of the actual players is much better than both the Markov agent and an agent with an *m*-step memory. This is not surprising, since it is likely that when nearing 253 hops, the long-term memory of the player also has a major influence. As discussed earlier, in the longer term, the player would probably have formed a route or some sort of survey knowledge; this may include the structure of the building, routes, and short cuts and, in general, means there is likely more structure to the mental map. We also observe that the Markov agent performs worse than the agent with perfect memory, regardless of the value of *m*. This is because the Markov agent has the same errors as the collective human memory, whereas the *m*-step agent has perfect memory.

### Expected hops given coverage

**Fig. 7:**
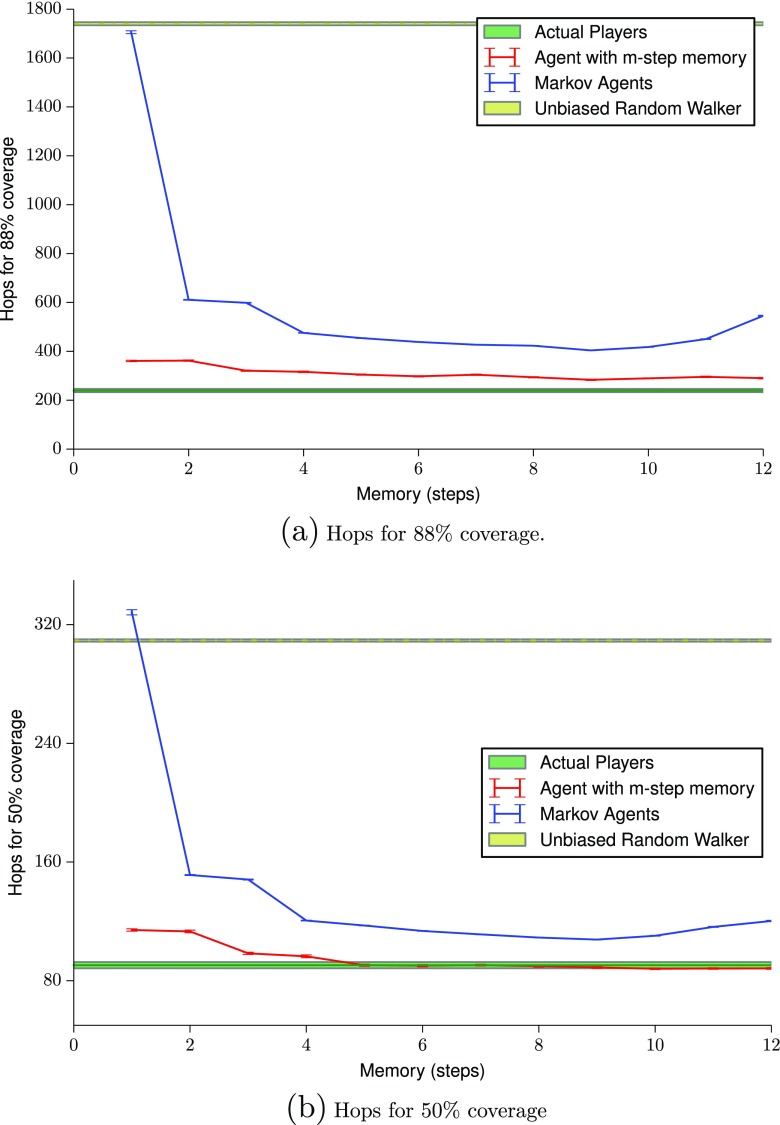
Standard error plot of the minimum number of hops required for obtaining given coverage and shows this as a function of memory size. As with Fig. 6, the low values of standard error on the agent paths are because these calculations are calculated over several thousand paths that are required for the radius of gyration to stabilize

We calculate the minimum number of hops required to obtain a given coverage. Unlike coverage, a hop count captures the dynamics of room revisits. The average final coverage for a player after the exploration phase of the game is (89±1)*%* as shown in Fig. 6. We first calculate the minimum number of hops required by the different agents to obtain this coverage; this is shown in Fig. 7a. The graph shows the same pattern as in Fig. 6. The only difference is that the number of hops required by the Markov agents increases again for large memory steps. This is probably because rather than going to new nodes, the Markov agent revisits old nodes; this results in the coverage not increasing.

As mentioned previously, the reason for the patterns observed might be the effect of long-term memory. In order to test this, the same expected number of hops for 50 % coverage is also determined and Fig. 7b is obtained. The magnitude of the difference between hops required for 50 % and 88 % shows a non linear increase, indicating that exploration becomes progressively more difficult. The figure also shows that agents with a memory of five or more steps seem to perform at the same level or better than humans. It is interesting to note that the performance of Markov agents is worse than agents with a simple *m*-step memory. Again, this is probably due to the imperfect nature of the short-term human memory on which it is based. The gap in performance between the Markov agent dataset and the actual player dataset is quite narrow, at *m*=7 to 9. This indicates that the room visited at any point can be reasonably predicted from the previous 6–8 rooms during this early phase of exploration.

### Empirical analysis

In this section, we conduct an empirical and qualitative analysis of the *actual player dataset*, i.e., the actions of the players at different locations. This analysis is intended to reveal the existence of patterns in exploration like definite decision points, the capability of the Markov agent to replicate these patterns, and the possible importance of cues in recognition and long-term memory, which were not discernible from the movement graphs discussed previously.

#### Existence of decision points

**Fig. 8:**
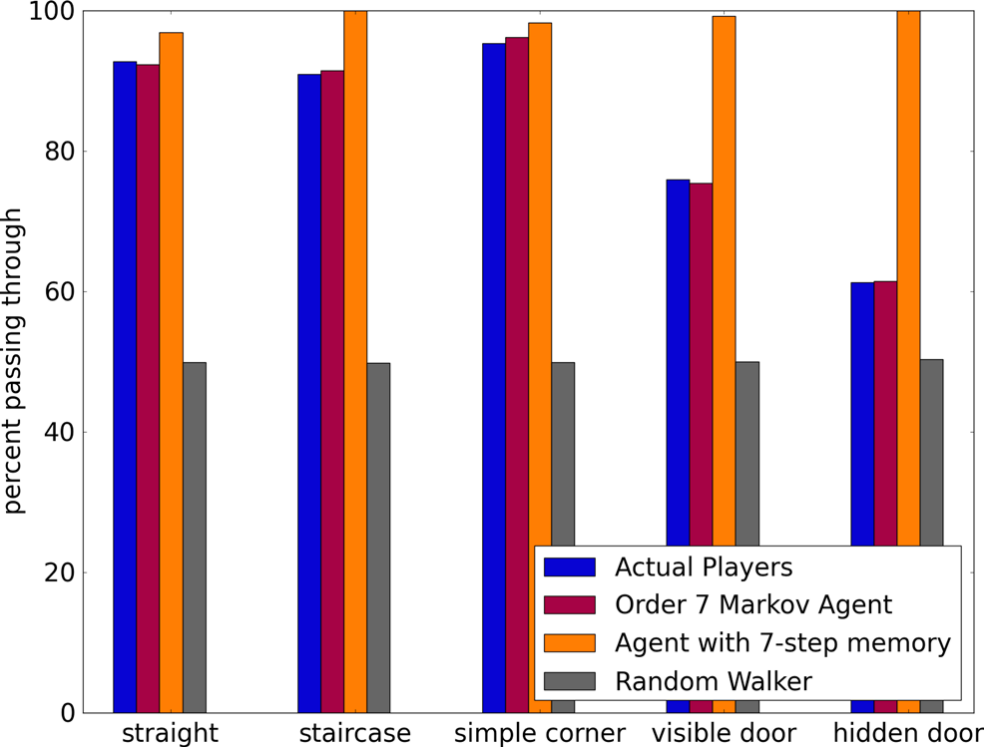
Chart summarizing the behavior of different kinds of agents at corridors of different types. Less than 10 % of the players make a conscious decision (i.e., change their direction) on a simple straight corridor (with no doors), a staircase, or even a simple corner. More interestingly, a little less than 25 % go back instead of opening a clearly visible door in front of them. However, when this next door is not clearly visible when they enter, 40 % of the players just go back through their entrance. This indicates that there are definite decision points during exploration. More interestingly, the 7^*th*^ order Markov agent exactly replicates this behavior while the other two agents’ behaviors significantly diverge

Figure 8 illustrates the decisions of people at different types of rooms and corridors, where it is possible for them to make a decision. At certain locations, such as *simple corridors* that have no rooms on the side (that is, they are simply connections between two areas), staircases and simple corners, the only decision that a player can make is whether to move forward or turn back. Turning back would require a conscious decision by the player. A pure random walker would have an equal chance of going back or forward. As shown in Fig. 8, the data reveals that players generally do not change their mind.

What is more interesting is the behavior of people at rooms that have just two doors. The data seems to reveal that if the opposite door is clearly visible from one door, then the room is used by the player almost exactly like a *simple corridor*, though there is a slightly higher chance of turning back. However, if the opposite door is not clearly visible when the person enters, i.e., it is at some angle to the view when the player enters or there is some furniture partially blocking the view to that door, then there is a more than 40 % chance of the person simply going back.

There is an argument to be made that this reluctance to move towards a slightly less visible door is a result of the input controls (keyboard and mouse) of the game. However, we believe this is not the case because the data that was used was observed from players who did not find it difficult to turn. Moreover, only the data of players who managed to complete the game within the time limit was included. As doing this required exploring a complicated three-floor environment with 44 rooms and plenty of turns, the players had to be able to use the movement and turning controls in a reliable and natural manner.

In order to demonstrate the ability of the Markov model to encapsulate factors other than memory that influence movement, we also plot the same behavior for a 7^*th*^-order Markov agent, a simple agent with seven-step memory and an unbiased random walker. As expected, the Markov agents reflect the same patterns as the actual player while the behavior of the other agents is completely different: the random walker makes no difference between going forward or back and the agent with memory never goes back.

#### Location recognition and memory

**Fig. 9:**
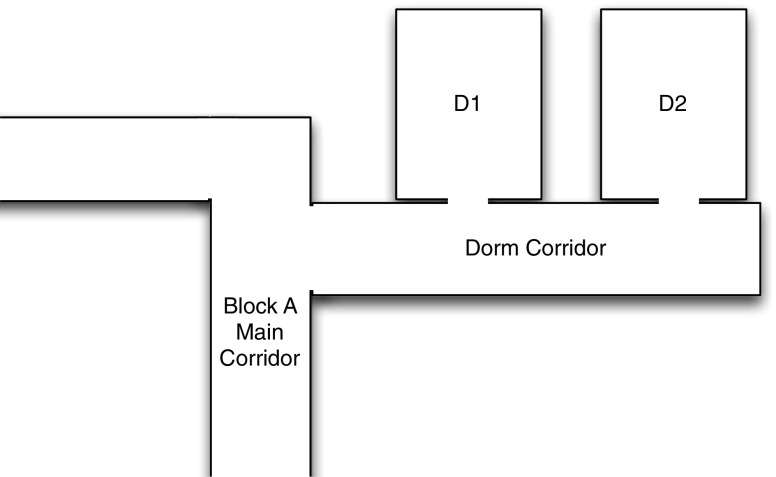
The layout of the area under consideration for analyzing location recognition and memory

**Fig. 10:**
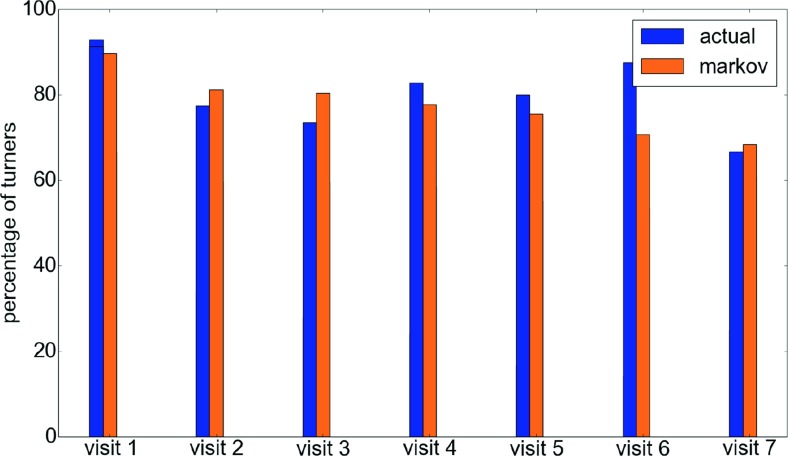
Movement of the players at the junction of Block A main corridor and the dorm corridor. As can be clearly seen, players keep revisiting the corridor despite having visited it before. The same pattern is seen in Markov agents too showing that only short-term memory is used

In the game environment, there exists a corridor that seems to reveal an interesting aspect of memory and exploration. The layout of this corridor is shown in Fig. 9. The corridor labeled *dorm corridor* is interesting because it is connected to the main Block A corridor only at one end^1^. The two rooms on this corridor (D1 and D2) do not have a checkpoint, a staircase, or any connections that make it at all relevant to the player. However, it lies on a commonly used corridor (marked Block A main corridor) and is used by most players at least once. In an ideal scenario, players would remember this fact and never visit the *dorm corridor* after the first visit to the junction of *Block A main corridor* and *dorm corridor*. The actual movement of the players at this junction is shown in Fig. 10. Surprisingly, the figure shows that during the course of the game, regardless of the number of times the junction is visited, players turn into the *dorm corridor* more often than not.

**Fig. 11:**
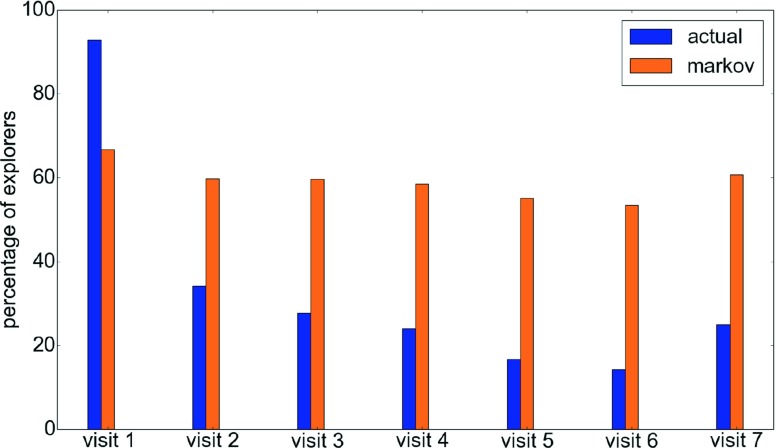
The behavior of the players after entering the *dorm corridor* during the knowledge testing phase. At this point, most players remember the corridor and head back to the main corridor without exploring. The fact that this is not seen in the Markov agents suggest that this is probably due to longer-term memory

At first, this leads to the conclusion that the players never learn and have no memory. However, a similar analysis of movement after entering the *dorm corridor* indicates that this is not the case. As shown in Fig. 11, about 80 % of the players head right back to the junction after entering this corridor. In order to understand why this happens, we can compare the player behavior against the Markov agent. We see that at the junction, the actual player movement is similar to the Markov agent movement, suggesting that the movement is primarily governed by short-term memory. However, in the dorm corridor, the actual players’ behavior is completely different from the Markov agent after the first visit, suggesting that long-term memory is being triggered by some factors at this point. This probably indicates that the context given by the location of signs and doors in the corridor helps the player remember the corridor, its location, and its use.

This could theoretically be verified through the continuous measurements of mouse movement trajectories of players at these points as in Spivey et al. (2005) and Freeman and Ambady (2010). However, this constant sampling and recording may have consequences on the frame-rate and playability of the game. Also, analyzing such information is complicated by the difficulty of matching mouse location with what exactly is seen on the screen as the player is constantly moving around a virtual world. However, this is definitely an avenue for future analysis.

## Conclusion and implications

In this paper, we presented a novel game-based methodology that allows for experimental investigation of human navigation and exploration. Although similar methodologies have been used to understand more general crowd behavior, we believe this is the first case in which quantitative analysis of a game has been used to understand the role memory plays in exploration. The novel Markovian analysis of the player’s movement in the game revealed a number of significant findings. Firstly, we showed that a simple memory model, with a depth of between 6 and 8, is sufficient to approximate a ‘human level’ of exploration efficiency. This was consistent in two measures of exploration efficiency: total coverage from a fixed number of hops and the number of hops required to obtain a fixed coverage. The memory depth of 6–8 seems to be consistent with well-known studies of human memory capacity (Miller, 1956). The experiments also highlighted the importance of junctions in the exploration process, in particular, decisions (that is, changing course) seem to almost exclusively occur at junctions. Explorers also try to reduce the number of decisions they have to make by proceeding to the next clearly visible room or corridor (assuming only one such room is visible). This pattern was also reflected in the Markov agents. Furthermore, the results showed that people seem to explore environments using a floor-first strategy, where they are reluctant to move to a different floor until they have finished exploring the current one. Finally, preliminary empirical analysis seems to suggest that easily recognizable locations probably help individuals improve exploration efficiency by enabling individuals to effectively remove sub-graphs of the room network from their cognitive map. However, verifying this final claim will require further testing either with mouse trajectory tracking or some other method of verifying the *heading* of the player.

Several of these observations could be made only through the analysis of the movement graphs and the Markovian analysis on the data. It would have been difficult to obtain this amount of data through the traditional experimental methodologies without significant cost in terms of time and effort. Thus, a game-based analysis is not only useful in making significant observations about human behavior but it also helps to open up new possibilities of research.

The simple agent-based memory model developed in this paper is shown to approximate human-like efficiency in its exploration strategy. We think this type of model is an excellent starting point for developing agent-based models that can be used to evaluate safety-by-design architecture in complex structures. We also see the experiments and methods presented here as a starting point for further investigations into the role of exploration and memory in human egress. Similar experiments could be conducted to evaluate the role of long-term memory in exploration, and perhaps validate the three-stage map building of Siegel and White (1975).

## Figures and Tables

**Fig. 12:**
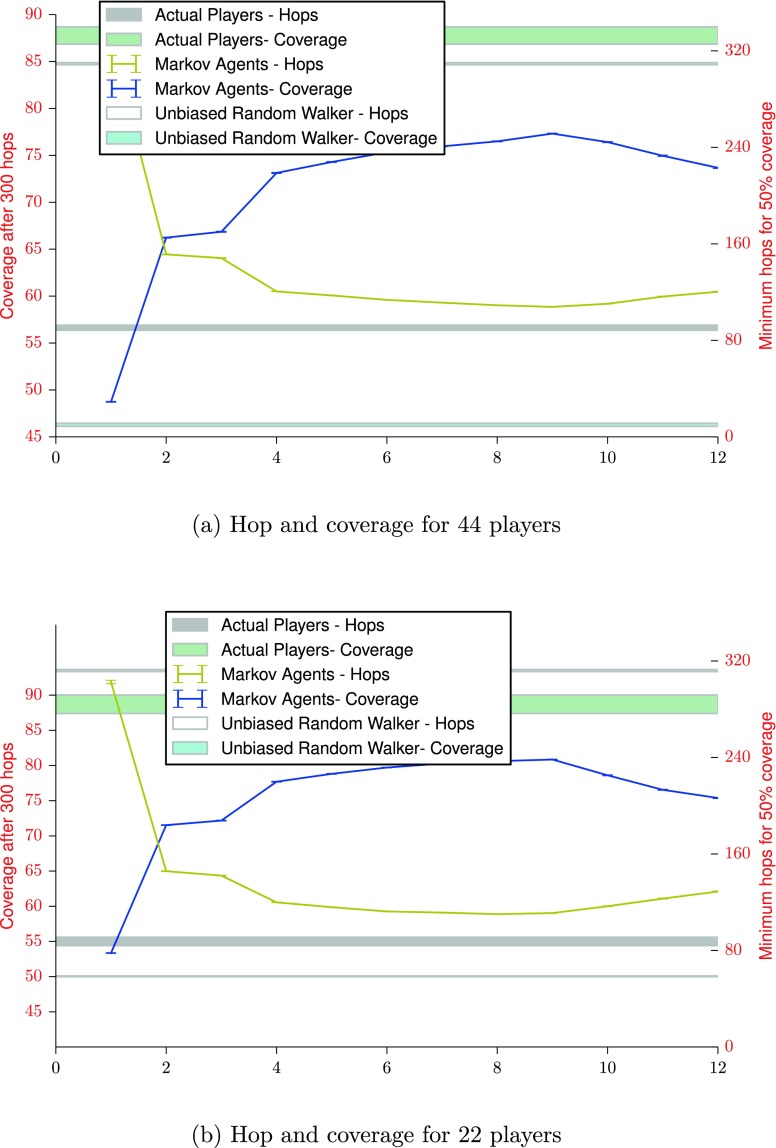
Hop and coverage graphs of different size of datasets seem to indicate that the peak is not dependent on data size

**Fig. 13:**
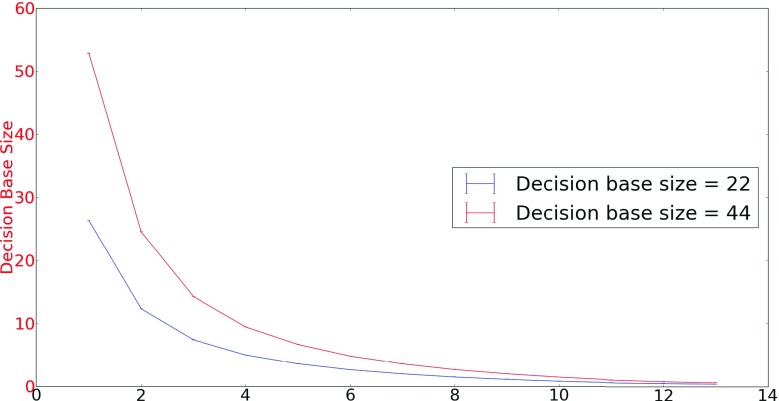
Comparison of the decision base sizes

**Fig. 14:**
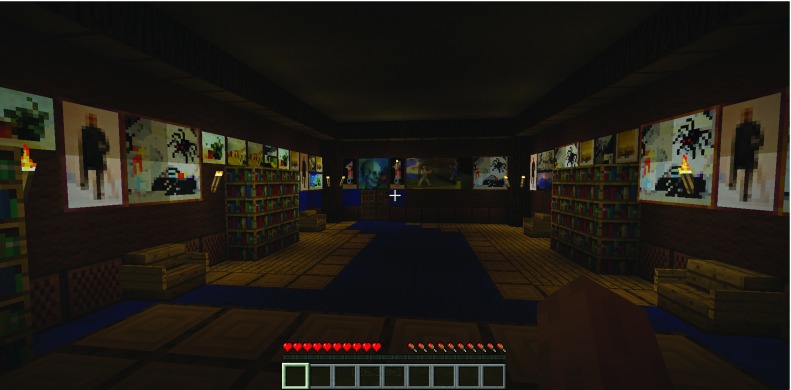
Picture gallery

## Appendix A: Validity check for Markov analysis

### Effect of dataset size

In order to check whether the peak in the hop and coverage data is a function of the dataset size, we hypothesized that if the peak does not change on doubling the dataset size, then the pattern that is seen is not an artefact of the dataset size. We plotted the same graph for *N*=22 (i.e., taking only 22 participants at random) and *N*=44 and checked if there is a shift in the peak to a higher value. As shown in Fig. 12, there is clearly no shift. The peak value still remains at 7–8 and starts dropping at 9.

### Decision base size at decision points

The Markov data calculation is based on aggregating the actions of the players at each decision point. The quality of the Markov data probability distribution calculated is thus based on the amount of data available to make predictions. In order to calculate an *m* ^*t**h*^-order probability of the next step, the calculations are done using the number of times the preceding sequence of *m* steps occurred and the choices made at that point by players in the dataset. Thus, as the frequency of said path decreases, there is lesser data for the probability calculation. The *m* ^*t**h*^-order Markov calculation becomes progressively less reliable and less different from random noise as *m* increases due to the limited size of the dataset.

To estimate the maximum value of *m* for which *m* ^*t**h*^ order calculations can be done reliably, we calculate the *decision base size* of the calculations. To calculate the decision base size for order *m*, we do the following: First, 30,000 paths of 300 hops are generated for an *m* ^*t**h*^-order Markov agent based on the dataset of size *N*. For each node in each path generated, the number of players that have actually made a decision at that point is counted. We divide this value by the number of decisions that are possible at that point. So a single decimal value is obtained for each node of each path generated. The average of this over all nodes a path gives the *path-specific decision base*. The average of these path-specific decision bases gives the decision base for the order *m* and dataset of size *N*. The result of this calculation is shown in Fig. 13.

The figure, firstly and most importantly, shows the usefulness of having a larger dataset in the Markov data calculation. Also, beyond the 9^*t**h*^ order, a decision base of size less than 2 is available, indicating that there are less than twice as many actions as there are choices on average at each decision point. This indicates that beyond the 9^*t**h*^ order the calculations are unreliable.

## Appendix B: Detailed description of the game

The premise of the game is that the player has been teleported into an abandoned palace where 11 people have been imprisoned in different locations spread across the three-floor environment. The objective of the game is for the player to free the 11 prisoners and subsequently follow instructions to open the main gate to the palace and escape. The palace is a three-floor building with 44 rooms. The layout of each floor of the palace is shown in Fig. 1. A player starts at the location indicated on the map with an X.

During the first few minutes of the game, the player is presented with the story line and interactively told how to use the controls and play the game through a set of signboards. They also follow a tutorial, which helps them free the first prisoner. Subsequently, the player is tasked to find and free the remaining ten prisoners. The locations of the prisons, as shown by the shaded areas in the map, are spread randomly around the building. This is the first knowledge-acquisition phase of the game and we call this the *exploration phase*. This phase requires the player to move around and explore the building. This phase can be reasonably equated to what a new visitor to a building (for example, a shopping mall) experiences.

The next phase, which we call *knowledge testing phase*, starts when the eleventh prisoner has been freed. During this phase, the cognitive map formed by the player is tested through a series of three tasks, which involve operating switches that were hidden during the exploration phase. By not revealing the nature of the second phase to the player at the beginning of the game and also by hiding the location of the knowledge testing tasks, we ensure that the player does not make a special effort in remembering locations which could artificially alter the cognitive map formed.

When the eleventh prisoner is freed and the exploration phase ends, the player is given instructions to proceed to the gallery room (shown in Fig. 14). This room would have been examined by the player during exploration. It is the only room in the palace whose walls are covered with paintings and this makes it reasonably likely that the player will remember this location because of its *perceptual salience* (Davis et al. 2009).

Once the player locates and presses the switch that is revealed in this room, the player is given instructions to move to the second-floor library. There are two important factors for choosing this particular location. Firstly, the library has four entrances and is very likely that the player would have entered this room multiple times during the exploration phase. Secondly, being on the second floor, there are multiple paths to this location from the gallery.

Once this location is found, the player is given the final instruction to proceed to their starting location to find the final switch that will open the main gate to the palace. Again, there are multiple routes to this location, some of which are significantly shorter than others. Also, being a starting location and in a somewhat central location, it is likely that it has been frequently visited and should have some *cognitive salience* (Davis et al. 2009).

We built the experiment inside the popular game Minecraft (Persson & Bergensten, 2009) in order to reduce the development effort and to allow for flexibility in environment creation.

### The Minecraft gaming environment

Minecraft^2^ is a Java-based multi-platform sandbox construction game. The game involves players creating and destroying various types of blocks in a first-person three-dimensional environment. In the original game, the player controls an avatar that can destroy or create blocks, forming buildings, structures, artwork, and even entire cities on multi-player servers or single-player worlds across multiple game modes. Players can break any block and build any block, provided he/she has the resources. Figure 14 gives a better idea of the look and feel of the game.

In the development of the game described in the previous section, we use an existing plug-in^3^ that constrains players so that they can only move around in the environment and interact with doors and switches, that is, elements that are essential for the experiment. A second modification is used^4^ to keep a log of the movements and actions of the players and store them in a MySql server for analysis. The player locations at different times, the time at which each prison was opened and the time taken to complete each task in the testing phase, are all recorded for this purpose.

Using Minecraft provided several advantages. Being a popular game with standard controls and a first-person view, enjoyability and playability are much easier to achieve. The first-person view and existing 3D models for avatars, doors, etc., ensures a degree of immersiveness that is generally more difficult to achieve. Another useful consequence of the popularity of Minecraft is the large community of developers and the number of Java-based plug-ins that are available for modifying the default Minecraft environment. This made it easier to develop the game and make the necessary modifications to make the experiment as required. However, the biggest advantage of using Minecraft is the ubiquity it offers, with over 35 million copies sold. If hosted on a publicly accessible server, it would theoretically not be difficult to get several hundred, if not a thousand, participants. Efforts that have been made towards this are presented later. In the context of immersion, it is also interesting to note the ongoing efforts to develop a version of Minecraft for Oculus Rift^5^, the upcoming commercial VR headset. This would open up exciting new possibilities for extension of the research presented here. In the present paper, we present the initial results of the game being played by participants on a local server.

This paper analyzed the result of 50 students playing the game on a local server. One of the limitations of the analysis discussed was the amount of data that was available for analysis, especially for the Markov analysis. The limited number of participants available also meant that it was difficult to test hypotheses on the task completion phase by creating and comparing the players’ movement in an alternate environment.

As discussed earlier, one of the big advantages of using a popular game like Minecraft is the ubiquity of the game. To make use of this ubiquity, we have hosted the game on a Linux server provided by Amazon Web Services and plan to collect data from this server over the next year. First, a simple web page^6^ was created where interested players can submit a request to play by using a registered Minecraft account. This request is sent to the Amazon Server. Python’s event-driven network engine *Twisted* (McKellar & Fettiq, 2013) was used to create a simple application that listens to these requests at a specified port and starts a white-listed Minecraft server to which only this particular user can connect. Once a request is successful, the player receives the IP address of the machine, which he proceeds to use to connect to the server and play the game. The Minecraft server is shut down once the player exits the game.

We hope to gather a much greater sample that may reveal more fundamental and interesting aspects of human exploration behavior than was possible to analyze with the limited dataset currently available.

## Footnotes

1 With reference to the previous discussion on decision points, it is important to remember that this is not a *simple corridor* because it has doors on the side and is thus a decision point.
